# Efzimfotase Alfa Improves Respiratory Capacity in Muscle Tissue From a Mouse Model of HPP

**DOI:** 10.1002/jmd2.70057

**Published:** 2025-12-31

**Authors:** Denise Devore, Juan Ruanova, Walter Voegtli, Derek Dunn, John Decker, Maurizio Mazzantini, Vincenzo De Tata, Maria Concetta Scavuzzo, Francesco Conti, Anna Petryk, Maria Luisa Brandi

**Affiliations:** ^1^ Pharmacology, Alexion, AstraZeneca Rare Disease Boston Massachusetts USA; ^2^ In Vitro and In Vivo Pharmacology, Alexion, AstraZeneca Rare Disease Boston Massachusetts USA; ^3^ Discovery Research, Alexion, AstraZeneca Rare Disease Boston Massachusetts USA; ^4^ Clinical Development, Alexion, AstraZeneca Rare Disease Boston Massachusetts USA; ^5^ MeTa+ Global SciComm, Alexion, AstraZeneca Rare Disease Boston Massachusetts USA; ^6^ Rheumatology Unit, Department of Clinical and Experimental Medicine University of Pisa Pisa Italy; ^7^ Department of Translational Research and New Technologies in Medicine and Surgery University of Pisa Pisa Italy; ^8^ C.I.M.E. Interdepartmental Center of Electron Microscopy University of Pisa Pisa Italy; ^9^ Department of Clinical and Molecular Medicine Sapienza University of Rome Rome Italy; ^10^ Global Medical Affairs, Alexion, AstraZeneca Rare Disease Boston Massachusetts USA; ^11^ FirmoLab, F.I.R.M.O. Italian Foundation for the Research on Bone Diseases Florence Italy

**Keywords:** efzimfotase alfa, enzyme replacement therapy, hypophosphatasia, mitochondrial dysfunction, muscle weakness, ultrastructural studies

## Abstract

Hypophosphatasia (HPP) is an inherited metabolic disease caused by deficient tissue‐nonspecific alkaline phosphatase (ALP) activity and characterized by skeletal and nonskeletal symptoms, including muscle weakness and fatigue. We hypothesized that mitochondrial respiration is impaired in muscle in HPP, independent of skeletal manifestations, and that the second‐generation ALP enzyme replacement therapy (ERT) efzimfotase alfa improves respiration. Akp2GW^−/−^ mice were used for this purpose. Body weight, bone mineralization, and survival were validated in Akp2GW^−/−^ mice versus Akp2^−/−^ mice, an established model of HPP. No significant differences were found, validating the Akp2GW^−/−^ model. Respiratory outcomes were measured in skeletal muscle fiber bundles in age‐ and sex‐matched Akp2GW^−/−^ and Akp2GW^+/+^ (wild‐type) mice; bone mineralization was assessed. Mean maximal respiration and mitochondrial spare respiratory capacity (SRC) in vehicle‐treated Akp2GW^−/−^ mice were 37% and 30% of values from wild‐type mice, respectively, independent of skeletal manifestations. Efzimfotase alfa treatment significantly improved maximal respiration in tissue from Akp2GW^−/−^ mice by 147% versus vehicle (*p* = 0.0059) and improved SRC by 262% versus vehicle (*p* = 0.0008). Mean maximal respiration and SRC in tissue from efzimfotase alfa‐treated Akp2GW^−/−^ mice were 92% and 107%, respectively, of tissue from wild‐type mice. Cellular ultrastructure of muscle biopsies from people with HPP showed atypical mitochondrial morphology, including branching cristae and dispersed matrix. In a mouse model of HPP, we show that the altered mitochondrial respiration in skeletal muscle is improved by ERT and that HPP is characterized by altered muscle mitochondrial morphology in humans. Together, these data suggest ERT could improve muscular symptoms in HPP.

## Key Points

- This article provides evidence for the presence of reduced mitochondrial respiratory capacity in a mouse model of hypophosphatasia (HPP), even in the absence of observable skeletal manifestations.
- Treatment with a second‐generation enzyme replacement therapy normalizes mitochondrial respiratory capacity in HPP mice.
- Altered mitochondrial morphology is demonstrated in micrographs of muscle tissue from patients with HPP, corroborating preclinical findings.

## 1 Introduction

Hypophosphatasia (HPP) is a rare inherited metabolic disorder caused by low activity of tissue‐nonspecific alkaline phosphatase (ALP) due to loss‐of‐function variants in *ALPL* (humans) and *Alpl*/*Akp2* (mice) [1]. Deficient ALP enzyme activity causes extracellular substrate accumulation, including inorganic pyrophosphate (PPi; potent inhibitor of bone mineralization); pyridoxal 5′‐phosphate (PLP; active form of vitamin B_6_); and phosphoethanolamine (PEA), which has an unclear biological function in HPP [2, 3].

Most skeletal manifestations of HPP, including rickets, bone deformities, fractures, and pseudofractures, are well characterized, easily documented with radiographs (except osteomalacia, which requires a bone biopsy), and linked to abnormal PPi metabolism [2, 4, 5, 6, 7, 8]. Among the many nonskeletal manifestations of HPP, one of the best documented is muscle weakness [5, 6, 7, 9]. However, the mechanisms underlying muscle weakness in HPP are not fully elucidated and may occur without overt skeletal symptoms [9], suggesting skeletal involvement cannot be the sole contributor.

*Akp2* knockout mice are often used to study HPP. They lack enzymatic activity, resulting in growth failure, seizures, and profound skeletal abnormalities commonly found in human infants with HPP [10, 11, 12]. Compared with wild‐type mice, Akp2^−/−^ mice demonstrate reduced grip strength, muscle fiber development, and motor coordination [13, 14]. Akp2^−/−^ mice also exhibit reduced complex I and II leak in the electron transport chain (ETC) relative to wild‐type mice, although uncoupled respiration was similar between groups [13]. In a large‐animal sheep model of HPP, which more closely parallels HPP in humans, muscle fibers varied in size and had incorrectly folded mitochondrial cristae [15]. Muscle from HPP sheep also had lower mitochondrial density and diminished oxidative phosphorylation [15, 16]. These data collectively suggest mitochondrial dysfunction may contribute to HPP muscle weakness.

In 2015, asfotase alfa, a tissue‐nonspecific ALP enzyme replacement therapy (ERT), was approved for HPP in the European Union, United States, and Japan [17, 18, 19, 20]. Asfotase alfa improves skeletal outcomes (e.g., rickets healing, fracture healing) and functional outcomes (e.g., walking ability, timed up‐and‐go test, chair rise test) in patients with HPP [8, 21, 22, 23, 24, 25, 26, 27], including those linked to improved muscle strength [8, 22, 25, 28, 29], although the mechanisms underlying this improvement are not well understood.

Efzimfotase alfa is a second‐generation ERT that is structurally related to asfotase alfa and currently under clinical investigation. Compared with asfotase alfa, efzimfotase alfa has several structural modifications, including the introduction of a point mutation (E108M) to increase enzyme activity and the removal of 2 N‐linked glycosylation sites to improve its pharmacokinetic profile and support less frequent dosing [30]. A first‐in‐human clinical trial demonstrated that, similar to asfotase alfa, efzimfotase alfa tends to normalize PPi levels in a dose‐dependent manner, suggesting its efficacy in improving bone mineralization [30]. Phase 3 studies with efzimfotase alfa are ongoing (NCT06079359, NCT06079281, NCT06079372).

The current translational studies document the structure of human sarcoplasmic mitochondria and explore the effects of efzimfotase alfa on respiratory outcomes in a murine model of HPP (Akp2GW^−/−^). The hypotheses were that (1) reduced respiratory capacity is present in skeletal muscle from Akp2GW^−/−^ mice, independent of skeletal mineralization, (2) efzimfotase alfa improves respiratory capacity in Akp2GW^−/−^ mice, and (3) mitochondrial alterations previously described in a sheep model of HPP are also present in muscle tissue from patients with HPP [15].

## 2 Methods

### 2.1 Measurements

Respirometry analysis was performed to determine whether mitochondrial dysregulation was present in muscle fiber bundles from AkpGW2^−/−^ mice versus age‐ and sex‐matched wild‐type mice. Spare respiratory capacity (SRC) is the difference between maximal and basal respiration in the ETC. SRC is a measure of the cellular ability to generate adenosine triphosphate (ATP) in response to increased energy demands that corresponds with mitochondrial fitness and the ability to adapt to stress conditions [31]. Reduced SRC is therefore indicative of mitochondrial dysfunction that becomes evident under periods of energetic stress [32]. SRC and other respirometry outcomes were measured in muscle fiber bundles from Akp2GW^−/−^ mice to show the effect of ALP enzymatic activity knockout.

Skeletal mineralization and morphology were assessed by radiography (Faxitron UltraFocus X‐Ray, Hologic, Marlborough, MA, USA) and microcomputed tomography (Quantum FX, PerkinElmer, Waltham, MA, USA). Images were analyzed with Quantum Ink (version 2.3.0.0) software for micro‐computed tomography‐generated images or VISION software for Faxitron X‐ray–generated images. Mineralization status was scored on a scale of 1–4, with 1 representing severe deficit (profound dysmorphology and complete absence of medial and distal phalange of the digits and complete lack of secondary ossification centers) and 4 representing unaffected bone (fully formed digits and all secondary ossification centers). Maximum tibia lengths were measured from X‐ray images using ImageJ software [33].

Transmission electron microscopy (TEM) was performed on muscle biopsies from patients with HPP to show the effect of ALP enzyme deficiency on muscle and mitochondrial morphology as surrogate markers of mitochondrial dysfunction.

### 2.2 Mouse and Human Study Ethics

All procedures associated with animal studies were carried out in accordance with Alexion, AstraZeneca Rare Disease Animal Use Protocols (AUP#917103‐002, AUP#14‐M061) and were approved by Alexion's Institutional Animal Care and Use Committee. Human studies were approved by the Ethics Committee of the Central Wide Area (Azienda Ospedaliero Universitaria Careggi, protocol number 2014/0025821, reference BIO 14.017) in Florence, Italy, and carried out based on guidelines of the International Conference on Harmonisation. Muscle biopsy samples were obtained after written consent was provided by patients to have their data reported anonymously.

### 2.3 HPP Animal Model Experiments

Animals were housed under conventional conditions at the Animal Care Facility, Alexion, AstraZeneca Rare Disease. Study breeders, nursing dams, pups, and weaned animals had free access to a commercial laboratory rodent diet supplemented with 325 ppm pyridoxine (Teklad Global 18% protein rodent diet; #TD.110088, Envigo, Indianapolis, IN). In the absence of pyridoxine supplementation, Akp2^−/−^ mice die at or before postnatal day (PND) 10 [11]; supplementation with 325 ppm pyridoxine extends the lifespan to PND 18 to 22 [12].

### 2.4 Akp2GW Model Validation

Outcomes were compared between Akp2GW mice and Akp2 mice to validate Akp2GW^−/−^ as a suitable model of HPP. Akp2GW^−/−^ mice were generated by genOWay SA (Lyon, France) using methodology described by Narisawa et al. to create an early generation sperm repository to avoid genetic drift [11]. Briefly, for Akp2^−/−^ mice, a neomycin resistance cassette was inserted into the EcoRV site of an Akp2‐KS vector, which contained a 6‐kb EcoRV‐BamHI fragment of the *Akp2* gene subcloned from 129 J mouse genomic DNA into the KS plasmid (Stratagene). For Akp2GW^−/−^ mice, the neomycin resistance cassette was inserted into exon 11 of the *Akp2* gene using a genomic fragment isolated from C57BL/6 mice via a LEM7‐HR vector. Both vectors contained a diphtheria toxin A cassette for negative selection. Targeting was performed in 129‐derived embryonic stem cell lines (for Akp2^−/−^ mice) or in C57BL/6‐derived embryonic stem cell lines (for Akp2^+/−^ mice) using homologous recombination. Embryonic stem cells were identified by polymerase chain reaction and Southern blot screening, selected for expansion, and subsequently injected into blastocysts. Cassette insertion resulted in complete loss of *Akp2* transcription; flanking regions and neighboring genes were not affected. Akp2GW^−/−^ mice are genetically identical to Akp2^−/−^ mice with respect to the *Akp2* deletion.

In vitro fertilization was performed at Jackson Laboratory (Bar Harbor, ME, USA) using Akp2GW^+/−^ sperm from 2 male founder mice and B6129S F1 oocytes (Stock #101043). In vitro fertilization was also performed using Akp2^+/−^ sperm from strain HPP‐M2h_F4 and B6129SF2/J oocytes (Stock #101045). Parental heterozygous mice (Akp2GW^+/−^ and Akp2^+/−^) were switched to a pyridoxine‐supplemented diet a minimum of 7 days prior to breeding. Akp2GW tail snips were acquired for genotyping between PND 1 and 3. Body weights of surviving mice were recorded throughout the study.

### 2.5 Akp2GW SRC Cohort

In vitro fertilization was performed at Jackson Laboratory using B6129S heterozygous Akp2GW^+/−^ cryopreserved sperm (Stock #404395) and B6129S F1 oocytes (Stock #101043) to obtain parental F0 mice that were subsequently bred to expand the colony to F2 and F3 generations. Akp2GW^−/−^ and Akp2GW^+/+^ study mice were obtained by breeding F2 and F3 heterozygous mice. Throughout the study, the Akp2GW^−/−^ mouse strain was maintained on a mixed genetic background. Tail snips were acquired between PND 1 and 3 for genotyping. Akp2GW^−/−^ mice were randomized to receive either 2 mg/kg efzimfotase alfa or phosphate‐buffered saline (vehicle) subcutaneously every other day (Q2D) from PND 3 to the end of study (PND 19–20 for vehicle‐treated mice or PND 19–31 for efzimfotase alfa‐treated mice). Pairs of age‐ and sex‐matched Akp2GW^+/+^ (wild‐type) and efzimfotase alfa‐ or vehicle‐treated Akp2GW^−/−^ mice were euthanized via isoflurane overdose followed by cervical dislocation. Muscle fiber bundles were immediately harvested from extensor digitorum longus (EDL) muscle and prepared for respirometry evaluation. Right hind limbs were dissected and frozen at −20°C until bone mineralization analysis was performed.

### 2.6 Respirometry Analysis

EDL muscle, including intact distal and proximal EDL tendons, was excised in Tyrode's solution, transferred to warm Dulbecco's Modified Eagle Medium (DMEM) supplemented with 2.5 mg/mL collagenase and 1% antibiotic‐antimycotic, and incubated at 37°C with 5% CO_2_ for at least 60 min. The muscle was then washed and gently pipetted up and down through a glass pipette until muscle fiber bundles were released. The wash and pipetting were performed with warm DMEM supplemented with 5 mg/mL recombinant human albumin, 25 mM HEPES pH 7.4, and 1% antibiotic‐antimycotic. A 96‐well Seahorse plate previously coated with mouse collagen IV and freshly coated with ice‐cold Matrigel was placed on a 37°C heat block. Immediately after placing the plate, EDL bundles containing 4 to 5 muscle fibers were carefully transferred and affixed to the center of the wells of the 96‐well Seahorse plate. Warm DMEM was added to the wells, and the plate was incubated at 37°C with 5% CO_2_ for 2 h. Wells were gently washed with Seahorse XF DMEM fortified to final concentrations of 10 mM glucose, 1 mM pyruvate, and 2 mM glutamine. Final volume in all wells was 180 μL. The plate was incubated at 37°C with 5% CO_2_ for 1 h. The mitochondrial oxygen consumption rate in EDL bundles was first measured at baseline and during the subsequent addition of 2.5 μM oligomycin, 0.5 μM carbonyl cyanide‐4 (trifluoromethoxy) phenylhydrazone, and 1 μM rotenone/antimycin A, which were dosed in series. The experiment was carried out using a Seahorse XFe96 bioanalyzer (Agilent Technologies, Santa Clara, CA).

### 2.7 Reagents

Efzimfotase alfa (95.9% purity) was synthesized at Alexion, AstraZeneca Rare Disease (New Haven, CT). DMEM, phosphate‐buffered saline without calcium or magnesium, HEPES (4‐(2‐hydroxyethyl)‐1‐piperazineethanesulfonic acid), and glutamine were obtained from Gibco/Thermo Fisher Scientific (Waltham, MA). Antibiotic‐antimycotic was purchased from Sigma‐Aldrich (St. Louis, MO). Materials for Seahorse, including cartridges, Mito Stress Test Kit, XF DMEM pH 7.4, glucose, and pyruvate, were purchased from Agilent Technologies. Collagen IV and Matrigel matrix were obtained from Corning (Corning, NY). Collagenase Type 2 was obtained from Worthington (Lakewood, NJ), recombinant human serum albumin was from ScienCell (Carlsbad, CA), and Tyrode's solution was from Boston BioProducts (Boston, MA).

### 2.8 Statistical Analysis of the Results in HPP Animal Models

Statistical analyses were performed on preclinical findings using GraphPad Prism version 9.3.1 (La Jolla, CA). A Kaplan–Meier plot of daily survival percentages was calculated for Akp2^−/−^ and Akp2GW^−/−^ strains, and differences were assessed with the log‐rank (Mantel‐Cox) test. Differences between or within Akp2 and Akp2GW mice were analyzed by the Mann–Whitney test when comparing two groups or 1‐way analysis of variance with Dunnet's multiple correction when comparing three or more groups. Analysis of respirometry data was performed using the Seahorse XF Cell Mito Stress Test Report Generator version 4.03 (Agilent Technologies). Oxygen consumption rate results were normalized to muscle fiber number and used to calculate the SRC. SRC in individual Akp2GW^−/−^ mice was expressed as a percentage of SRC in age‐ and sex‐matched wild‐type mice. Differences between vehicle‐treated and efzimfotase alfa‐treated Akp2GW^−/−^ mice were analyzed using the 1‐tailed Mann–Whitney test.

### 2.9 TEM Analysis of Patients' Biopsy Samples

An analysis using TEM was performed on muscle biopsy samples derived from 3 patients with HPP (2 of whom were twin sisters) and 1 healthy volunteer to further corroborate preclinical findings. Muscle tissue samples were fixed with 2.5% (vol./vol.) glutaraldehyde in 0.1 mmol/L cacodylate buffer, pH 7.4 for 1 h at 4°C, and then postfixed in 1% (vol./vol.) cacodylate‐buffered osmium tetroxide for 2 h at room temperature. Samples that were dehydrated in a graded series of ethanol, transferred to propylene oxide, and embedded in Epon‐Araldite. Ultrathin sections (60–80 nm thick) were cut with a diamond knife, placed on formvar/carbon‐coated copper grids (200 mesh), stained with uranyl acetate and lead citrate, and observed under a Jeol 100 SX transmission electron microscope or a Jeol JEM‐F200 transmission electron microscope.

## 3 Results

### 3.1 Mouse Model Results

#### 3.1.1 Akp2GW ^−/−^ Model Validation

**FIGURE 1:**
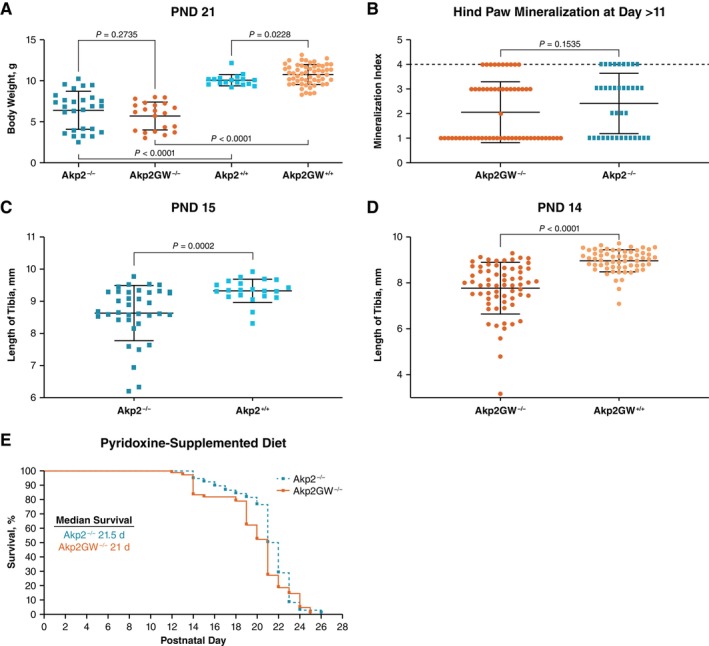
Comparison of HPP outcomes in Akp2GW and Akp2 mouse strains. (A) Body weight analysis at PND 21 in Akp2GW and Akp2 mice. (B) Hind paw bone mineralization analysis in Akp2GW^−/−^ and Akp2^−/−^ mice. (C, D) Tibia length measurement analysis in homozygous knockout mice versus heterozygous and wild‐type mice. (E) Kaplan–Meier survival plot in Akp2^−/−^ and Akp2GW^−/−^ mice. HPP, hypophosphatasia; PND, postnatal day.

Mean body weights at PND 21 were not significantly different between Akp2GW^−/−^ mice and Akp2^−/−^ mice (*p* = 0.2735); however, both groups had lower body weights compared with their wild‐type counterparts (Figure 1A). Body weight was not different between Akp2GW^+/−^ and Akp2^+/−^ mice, and body weights in heterozygous mice were similar to those of wild‐type mice of either genotype (data not shown). Hind paw mineralization at PND > 11 was not significantly different between Akp2GW^−/−^ and Akp2^−/−^ mice (*p* = 0.1535) (Figure 1B). For both Akp2GW and Akp2 mice, knockout mice had significantly shorter tibial lengths compared with wild‐type mice at PND 14 and PND 15, respectively (Figure 1C,D). Tibial length was similar between heterozygous and wild‐type mice in both models (data not shown). Median survival times from birth were similar between Akp2GW^−/−^ mice and Akp2^−/−^ mice (*p* = 0.1416), and no mice from either group survived beyond PND 26 (Figure 1E). As such, Akp2GW^−/−^ mice phenocopy survival and bone mineralization of Akp2^−/−^ mice and are an appropriate model of HPP in infants with life‐threatening disease.

#### 3.1.2 Respirometry

**FIGURE 2:**
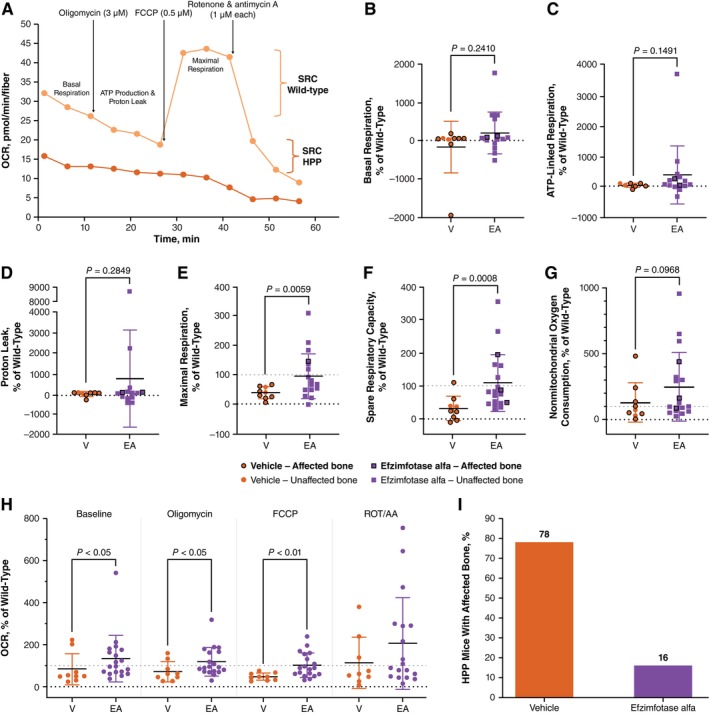
Bone and respirometry analysis in EDL muscle fiber bundles from Akp2GW mice. Akp2GW^−/−^ mice were treated with vehicle or efzimfotase alfa (2 mg/kg) Q2D from PND 3 to end of study (PND 19–31). (A) Representative respirometry plot from single Akp2GW^−/−^ and AkpGW^+/+^ (WT) mice. (B–G) Respirometry endpoints and (H) bioenergetic signature in isolated EDL muscle fiber bundles from vehicle‐treated (*n* = 9) and efzimfotase alfa‐treated (*n* = 19) Akp2GW^−/−^ mice expressed relative to age‐ and sex‐matched WT mice. Horizontal line indicates mean; error bars indicate standard deviation. (I) Analysis of hind paw bone mineralization in vehicle‐treated (*n* = 9) and efzimfotase alfa‐treated (*n* = 19) Akp2GW^−/−^ mice. AA, antimycin A; ATP, adenosine triphosphate; EA, efzimfotase alfa; EDL, extensor digitorum longus; FCCP, carbonyl cyanide‐4 (trifluoromethoxy) phenylhydrazone; HPP; hypophosphatasia; OCR, oxygen consumption rate; PND, postnatal day; Q2D, every other day; ROT, rotenone; V, vehicle; WT, wild‐type.

Respirometry results were stratified by affected or unaffected skeletal mineralization. A representative plot of the Mito Stress Test comparing wild‐type and Akp2GW^−/−^ mice is shown in Figure 2A. Mean basal respiration, ATP‐linked respiration, and proton leak were not significantly different between vehicle‐treated and efzimfotase alfa‐treated Akp2GW^−/−^ mice; values from these measures were similar to those of untreated wild‐type mice (Figure 2B–D).

Mean maximal respiration in muscle fiber bundles from vehicle‐treated Akp2GW^−/−^ mice was 37% of the maximal respiration in untreated wild‐type mice (Figure 2E). Maximal respiration values were depleted in vehicle‐treated Akp2GW^−/−^ mice relative to wild‐type mice, regardless of whether the mice had affected (undermineralized) bone (Figure 2E). Efzimfotase alfa treatment in Akp2GW^−/−^ mice significantly increased maximal respiration by 147% relative to vehicle treatment, and mean maximal respiration in efzimfotase alfa‐treated mice was 92% of the maximal respiration in untreated wild‐type mice (Figure 2E). Three efzimfotase‐alfa treated mice still had affected (undermineralized) bone at the time of assessment; maximal respiration in these mice was 67%, 70%, and 143% of the SRC of wild‐type control mice (Figure 2E).

The mean SRC of muscle fiber bundles from vehicle‐treated Akp2GW^−/−^ mice was 30% of SRC in untreated wild‐type mice (Figure 2F). Two vehicle‐treated Akp2GW^−/−^ mice had unaffected bone; SRC values of muscle fiber bundles in these mice were 35% and 37% of their wild‐type counterparts. Treatment with efzimfotase alfa significantly increased muscle SRC by 262% relative to vehicle‐treated Akp2GW^−/−^ mice (*p* = 0.0008) (Figure 2F). Mean SRC in efzimfotase alfa‐treated Akp2GW^−/−^ mice was similar to that of wild‐type mice (107%) (Figure 2F). SRC in mice with affected bone was 48%, 86%, and 194% of the SRC of wild‐type control mice. There was no significant difference between mean nonmitochondrial oxygen consumption rates in muscle fiber bundles from vehicle‐treated versus efzimfotase alfa‐treated Akp2GW^−/−^ mice (Figure 2G). Efzimfotase alfa treatment significantly increased the oxygen consumption rate relative to vehicle treatment in Akp2GW^−/−^ mice at baseline and after the addition of oligomycin and carbonyl cyanide‐4 (trifluoromethoxy) phenylhydrazone but not after the addition of rotenone/antimycin A (Figure 2H).

#### 3.1.3 Bone Analysis in Akp2GW Mice Assayed for SRC

X‐ray analysis of the hind paw of treated and untreated Akp2GW^−/−^ mice was performed to determine whether treatment with efzimfotase alfa improved bone mineralization and to determine whether the effect of ERT on maximal respiration and SRC is independent of bone. Consistent with results shown in Figure 1B, bone mineralization was unaffected in 22% of vehicle‐treated Akp2GW^−/−^, despite *Akp2* knockout (Figure 2I). Efzimfotase alfa improved the bone mineralization status in 84% of Akp2GW^−/−^ mice (Figure 2I).

### 3.2 Human Muscle Biopsy Results

**FIGURE 3:**
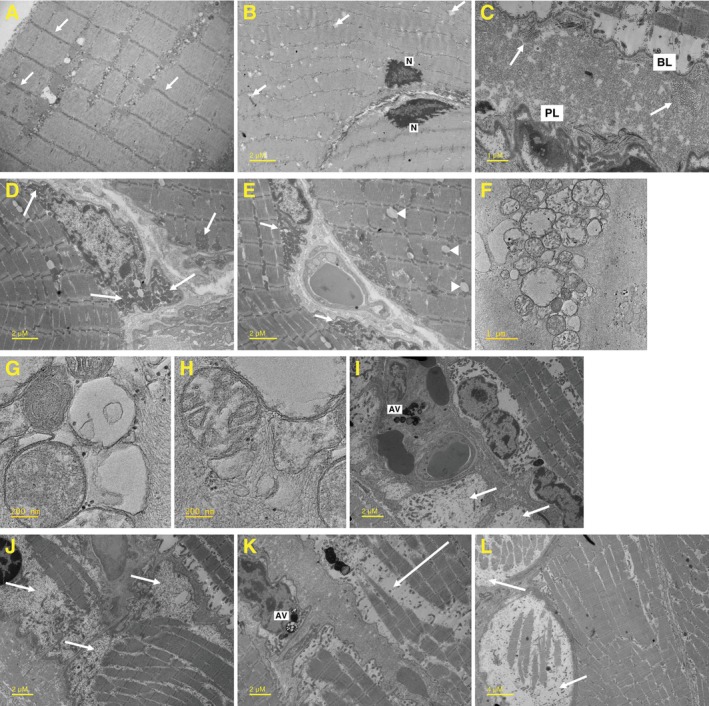
Ultrastructure of skeletal muscle from patients with HPP. Representative TEM images of skeletal muscle from (A) a healthy volunteer and (B–K) patients with HPP. (A) Arrows indicate skeletal muscle z‐lines; (B) arrows indicate sarcoplasmic reticulum dilations; (C) arrows indicate glycogen; (D) labels indicate perinuclear and subsarcolemmal aggregates of swollen mitochondria; (E) arrows indicate large perinuclear aggregates of mitochondria, arrowheads indicate lipid droplets; (F–H) panels show higher magnification of mitochondrial alterations (fragmented and branching cristae and dispersed matrix); (I–L) arrows indicate myofibril loss. AV, autophagic vacuoles; BL, basal lamina; HPP, hypophosphatasia; N, nucleus; PL, plasmalemma; TEM, transmission electron micrography.

TEM was used to visualize mitochondrial morphology as a surrogate marker of mitochondrial dysfunction in muscle biopsy samples from 3 patients with HPP and a healthy volunteer. An example of the normal ultrastructure of human skeletal muscle is shown in Figure 3A, in which normal skeletal muscle z‐lines are visible. In muscle cells from patients with HPP, nuclei with thickened and highly electron‐dense chromatin are often peripherally located and the cytoplasm appeared reduced (Figure 3B). Accumulation of glycogen and separation of the basal lamina from the plasma membrane were observed (Figure 3C). Mitochondria often appear quite dilated and frequently tend to form aggregates with subsarcolemmal or perinuclear localization (Figure 3D,E). Higher magnification details of mitochondrial alterations (fragmented and branching cristae and dispersed matrix) are shown in Figure 3F–H. Focal or more extensive areas of myofibril loss are shown in Figure 3I–L.

## 4 Discussion

The results of this study provide evidence of reduced respiratory capacity in a mouse model of HPP, which was improved by efzimfotase alfa treatment. Notably, the improvement in respiratory capacity after efzimfotase alfa treatment occurred independently of its effects on bone mineralization. TEM analysis of muscle biopsy samples derived from patients with HPP showed myofibril loss and alterations in mitochondrial microstructure. These data collectively suggest that mitochondrial dysfunction may be present in HPP and highlight the potential of ERT to improve mitochondrial respiration.

In the current study, muscle tissue from vehicle‐treated HPP mice had lower maximal respiration and SRC relative to tissue from wild‐type mice, indicating that the electron transport chain has less ability to consume oxygen under stress in HPP, which may affect ATP production. In contrast, basal respiration, ATP‐linked respiration, proton leak, and nonmitochondrial oxygen consumption were similar between wild‐type mice and vehicle‐treated HPP mice. Collectively, these findings indicate that muscle tissue from vehicle‐treated HPP mice has mitochondrial metabolism similar to that of wild‐type mice, except in times of energetic stress. In addition, efzimfotase alfa treatment affected only maximal respiration and SRC in muscle tissue from HPP mice while having no effect on other respirometry parameters, suggesting that the compound has minimal off‐target effects on basic mitochondrial processes.

SRC is defined as the difference between maximal and basal respiration. Inspection of the data (not shown) from individual muscle fiber bundles reveals that SRC and maximal respiration do not consistently exhibit a proportional relationship. There were instances where individual tissue samples from treated animals exhibited elevated maximal respiration without a corresponding increase in SRC, and vice versa, reflecting variability in basal respiration. This lack of direct correlation underscores the importance of independently assessing SRC and maximal respiration, as each parameter provides distinct insight into mitochondrial function and adaptability. Overall, the reduced respiratory capacity observed in vehicle‐treated HPP mice in the current study suggests that non‐treated HPP mice have impaired mitochondrial adaptability within muscle fibers.

The reduced mitochondrial adaptability may be due to limited pools of intracellular PLP, which are thought to occur due to HPP [2, 34, 35]. PLP is required for the assembly of iron–sulfur clusters, which are required for the operation of the tricarboxylic acid cycle and the ETC by transporting electrons through complexes I, II, and III to complex IV [36, 37, 38]. Impaired iron–sulfur cluster biogenesis can cause hypotonia and muscle weakness [39]. PLP is also required for the biosynthesis of the ETC component coenzyme Q_10_, which is necessary for mitochondrial ATP production [40]. Finally, PLP is an obligatory cofactor for the synthesis of 5‐aminolevulinic acid, a precursor of heme synthesis [41, 42]. Heme plays a central role in oxidative phosphorylation and oxygen consumption in the ETC, predominantly in the cytochromes where the heme iron interconverts between its oxidation states, thereby allowing the transfer of electrons [43]. Disruption of these PLP‐dependent mechanisms may cause dysregulation of the ETC and potentially contribute to dysregulated mitochondrial respiration observed in the current study.

TEM findings from the current study are consistent with findings from a sheep model of HPP and may suggest dysregulation of PLP‐dependent metabolism. Glycogen phosphorylase, the enzyme catalyzing the first step in glycogenolysis, is PLP‐dependent [44]. Thus, intracellular PLP deficiency in HPP may lead to the accumulation of glycogen in the cytosol, which was present in TEM images from the present study. Incorrectly folded mitochondrial cristae were reported in muscle cells from a sheep model of HPP and were associated with reduced mitochondrial density and oxidative phosphorylation [15, 16]. The myofibril loss seen in the current study is in agreement with an independent study that reported reduced myofibril diameter in 2 participants with HPP and myopathy [45]. Previous studies showed that treatment with asfotase alfa improves muscle strength as early as 6 months after initiating ERT, suggesting the mechanism underlying muscle weakness in HPP is reversible [8, 25, 28].

The current study has several limitations. While the data presented herein demonstrate differences in maximal respiration and SRC by genotype and ERT, direct measurement of ETC enzymes and in situ enzymatic staining of ETC activity in muscle samples was not performed, thus limiting interpretation of the mechanism underlying these results. No additional analyses of the muscle from Akp2GW mice, including TEM or Western blotting for mitochondrial fusion or fission proteins, were performed. TEM was performed on muscle tissue from 3 patients with HPP, limiting the generalizability of the results. Furthermore, while the discussed PLP‐mediated mechanisms of mitochondrial dysfunction are conceivable, they remain speculative and need further examination. It is also possible that the mitochondrial alterations and ultrastructural observations reported in this analysis are due to other mechanisms. Finally, no functional assessments of muscle, such as grip strength, were performed in Akp2GW^−/−^ mice, thus limiting the functional interpretation of respiratory outcomes. Further work is required to understand the mechanisms underlying reduced respiratory capacity in HPP and to potentially link the changes reported in this study to disease manifestations.

## 5 Conclusion

This study demonstrates reduced respiratory capacity and the efficacy of efzimfotase alfa at restoring respiratory capacity in a mouse model of HPP. Further, this study validates Akp2GW^−/−^ mice as an acceptable model to study HPP. Muscle tissue samples from patients with HPP demonstrate alterations in muscle fibers and mitochondrial morphology. Overall, the results of this study suggest that ERT may improve mitochondrial respiratory capacity in patients with HPP independent of its effects on bone mineralization. Potential mechanisms connecting reduced respiratory capacity and the development of nonskeletal manifestations of HPP require further investigation.

## Author Contributions

Study design: D. Devore, V. De Tata, Maria Concetta Scavuzzo. Collection and assembly of data: J. Ruanova, D. Devore, V. De Tata, Maria Concetta Scavuzzo, M. Mazzantini. Data analysis: D. Devore, V. De Tata, Maria Concetta Scavuzzo. Data interpretation: All authors. Manuscript review and revisions: All authors. Final approval of manuscript: All authors. Guarantor: M.L. Brandi.

## Funding

This study was sponsored by Alexion, AstraZeneca Rare Disease (Boston, MA, USA). Editorial support was provided by Peloton Advantage, LLC, an OPEN Health company, funded by Alexion, AstraZeneca Rare Disease.

## Ethics Statement

All procedures associated with this study were carried out in accordance with Alexion, AstraZeneca Rare Disease Animal Use Protocols (AUP#917103‐002, AUP#14‐M061), and were approved by Alexion's Institutional Animal Care and Use Committee (100 College St, New Haven, CT 06510). All institutional guidelines for the care and use of laboratory animals were followed.

## Conflicts of Interest

The authors declare that the research was conducted in the absence of any commercial or financial relationships that could be construed as a potential conflicts of interest. Denise Devore, Juan Ruanova, Walter Voegtli, Derek Dunn, John Decker, and Anna Petryk are/were employees of Alexion, AstraZeneca Rare Disease, and may hold stock/stock options in Astra/Zeneca. Francesco Conti has received speakerships from and has consulted for Alexion. Maurizio Mazzantini, Vincenzo De Tata, and Maria Concetta Scavuzzo have nothing to disclose. Maria Luisa Brandi received honoraria from Amgen, Ascendis, Bruno Farmaceutici, Calcilytix, and Kyowa Kirin; grants and/or speakerships from Alexion, Amgen, Amolyt, Bruno Farmaceutici, CoGeDi, Echolight, Gedeon Richter, Kyowa Kirin, Monte Rosa Therapeutics, and UCB; and has consulted for Aboca, Alexion, Amolyt, Bruno Farmaceutici, Calcilytix, Echolight, EnteraBio, Kyowa Kirin, Personal Genomics, and Septerna.

## Supporting information


**Data S1:** Supporting Information.

## Data Availability

All data supporting the findings of this study are available from the corresponding author upon reasonable request.
