# Supplementary material for: Efzimfotase Alfa Improves Respiratory Capacity in Muscle Tissue From a Mouse Model of HPP
Source: JIMD Rep. 2025 Dec 31;67(1):e70057. doi: 10.1002/jmd2.70057 (PMC12755057; doi:10.1002/jmd2.70057)
Supplement: Supplementary file 1 — Data S1: Supporting Information. [file JMD2-67-e70057-s001.docx]

**SUPPLEMENTAL MATERIAL**

**PLAIN LANGUAGE SUMMARY**

*The first draft of the Plain Language Summary was generated using a generative AI model that distills complex scientific publications into plain language summaries. As part of AZ's commitment to innovation and ethical AI use, this summary was generated within the secure confines of AZ's infrastructure, ensuring both data privacy and adherence to copyright laws. The authors are accountable for the accuracy and integrity of the final draft of the Plain Language Summary.*

We conducted this research to understand why patients with Hypophosphatasia (HPP), a rare disease caused by low activity of an enzyme called tissue-nonspecific alkaline phosphatase (ALP), experience muscle weakness independent of bone involvement. We suspected that the muscles might have reduced energy production issues due to mitochondrial dysfunction. Mitochondria are parts of the cell that generate energy through a process called oxidative phosphorylation.

We used a mouse model that represents HPP to study muscle energy production. We compared mice with HPP to healthy mice by measuring a key indicator of mitochondrial function known as spare respiratory capacity (SRC) in muscle tissues. We also treated the HPP mice with efzimfotase alfa, which is designed to replace the deficient ALP enzyme. Additionally, we examined muscle tissue from HPP patients using electron microscopy to observe the high-resolution structure of the muscle and mitochondria.

We found that the HPP mice had significantly lower SRC in their muscles, which means their mitochondria were not producing energy efficiently. However, after treatment with efzimfotase alfa, their SRC improved dramatically, to slightly above the level of healthy mice. The muscle tissues from HPP patients also showed abnormal mitochondrial structures, consistent with mitochondrial dysfunction in HPP. These results suggest that muscle weakness in HPP patients might be due to problems with energy production in their muscles due to mitochondrial dysfunction, and that efzimfotase alfa could potentially help improve their muscle function.

For more detailed information, readers can refer to the original scientific publication or consult medical databases such as PubMed for related studies on Hypophosphatasia, mitochondrial dysfunction, and enzyme replacement like efzimfotase alfa.
